# Therapeutic strategies and predictive models for Xp11.2 translocation/TFE3 gene fusion renal cell carcinoma in adults based on data of two Chinese medical centers

**DOI:** 10.18632/aging.205452

**Published:** 2024-01-22

**Authors:** Yunkai Yang, Changfeng Zhao, Zhida Wang, Feng Liu, Ming Zhao, Huiwen Yang, Jun Chen, Xuejing Chen, Min Shi, Dixing Jiang, Xiaoting Luo, Yue Duan, Yuchen Bai

**Affiliations:** 1 Department of Urology, Urology and Nephrology Center, Zhejiang Provincial People’s Hospital, Hangzhou Medical College, Hangzhou, Zhejiang 310011, China; 2 Graduate School of Bengbu Medical College, Bengbu, Anhui 233030, China; 3 Department of Clinical Laboratory, Zhejiang Provincial People’s Hospital, Hangzhou, Zhejiang 310011, China; 4 Department of Thoracic Surgery, The Second Affiliated Hospital of Zhejiang Chinese Medical University, Hangzhou, Zhejiang 310005, China; 5 Department of Urology, The Second Affiliated Hospital of Zhejiang Chinese Medical University, Hangzhou, Zhejiang 310005, China; 6 Department of Clinical Laboratory, The Second Affiliated Hospital of Zhejiang Chinese Medical University, Hangzhou, Zhejiang 310005, China; 7 Department of Medical Psychology, The Second Affiliated Hospital of Zhejiang Chinese Medical University, Hangzhou, Zhejiang 310005, China; 8 Department of Urology, Zhejiang Medical and Health Group Hangzhou Hospital of Hangzhou Medical College, Hangzhou, Zhejiang 310022, China

**Keywords:** rare cancer, TFE3-RCC, Xp11.2 translocation, predict, prognosis

## Abstract

Objective: This study aims to establish an effective predictive model for predicting Xp11.2 translocation/TFE3 gene fusion renal cell carcinoma (TFE3-RCC) and develop optimal therapeutic strategies.

Methods: Data from 4961 patients diagnosed with renal cell carcinoma at two medical centers in China were retrospectively analyzed. A cohort of 1571 patients from Zhejiang Provincial People's Hospital (Ra cohort) was selected to construct the model. Another cohort of 1124 patients from the Second Affiliated Hospital of Zhejiang Chinese Medical University was used for external validation (the Ha cohort). All patients with TFE3-RCC in both cohorts were included in the Ta cohort for the prognostic analysis. Univariate and multivariate binary logistic regression analyses were performed to identify independent predictors of the predictive nomogram. The apparent performance of the model was validated. Decision curve analysis was also performed to assess the clinical utility of the developed model. Factors associated with progression and prognosis in the Ta cohort were analyzed using the log-rank method, and Cox regression analysis and Kaplan-Meier survival curves were used to describe the effects of factors on prognosis and progression.

Results: Univariate and multivariate logistic regression analyses demonstrated that age, sex, BMI, smoking, eosinophils, and LDL were independent predictors of TFE3-RCC. Therefore, a predictive nomogram for TFE3-RCC, which had good discriminatory power (AUC = 0.796), was constructed. External validation (AUC = 0.806) also revealed good predictive ability. The calibration curves displayed good consistency between the predicted and observed incidences of TFE3-RCC. Invasion of regional lymph nodes, tyrosine kinase inhibitors, and surgical methods were independent factors associated with progression. Tyrosine kinase inhibitors are independent prognostic factors.

Conclusion: This study not only proposed a high-precision clinical prediction model composed of various variables for the early diagnosis of Xp11.2 translocation/TFE3 gene fusion renal cell carcinoma but also optimized therapeutic strategies through prognostic analysis.

## INTRODUCTION

Xp11.2 translocation/TFE3 gene fusion renal cell carcinoma (TFE3-RCC) is recognized as a distinct pathological tumor. In 2004, the World Health Organization (WHO) classified it as an independent subtype of renal cancer [1]. A few TFE3 and TFEB expressions are involved in the physiological regulation of normal endosomes. However, TFE3 gene breaks down at Xp11.2, and equilibrium translocations with ASPL, PSF, and other genes form new fusion genes, contributing to TFE3 or TFEB fusion gene formation and their high expression *in vivo*, thus causing renal cell carcinoma [2–6]. According to the WHO classification of renal cell carcinoma in 2016, it belongs to the MiT family of translocated renal cancers. Clinical studies have revealed that they account for at least a third of pediatric kidney cancers, with only a small number of adult cases reported. The TFE3-RCC incidence in adults is very low, accounting for approximately 0.9%–4% of renal cell carcinoma cases [2, 7–10]. The number of TFE3-RCC cases in adults may far exceed that in pediatric patients, although they may be uncommon in percentage. This is because RCC is more prevalent in adults than in children.

TFE3-RCC was formerly believed to be rather indolent even when diagnosed at advanced stages [11, 12], but some studies of aggressive clinical cases in adults have been reported recently [13–15]. Moreover, our cognition of the risk factors and treatment of TFE3-RCC is incomplete, resulting in a lack of clear concepts for diagnosing and developing individualized treatment. Most TFE3-RCC patients have no systematic treatment, resulting in a poor prognosis. Therefore, clinical issues today include how to predict TFE3-RCC before surgery and what therapy adult patients should choose. This study aimed to develop and validate predictive models for TFE3-RCC in two large medical centers in China, exploring the optimal treatment for TFE3-RCC through comparative treatment regimens and prognostic analysis.

## MATERIALS AND METHODS

### Study design and participants

This dual-center retrospective study involved RCa patients from two independent regional medical centers in Zhejiang, China. Data for 2520 patients admitted to Zhejiang Provincial People’s Hospital from 2008-02 to 2023-05 were collected and labeled as the R cohort for nomogram construction. Then, data for 2041 patients admitted to the Second Affiliated Hospital of Zhejiang Chinese Medical University from 2005-05 to 2023-04 were collected, labeled as the H cohort, and used for validation.

Furthermore, 149 TFE3-RCC patients were identified from data from two medical centers and labeled as the T-cohort, which was used for prognostic analysis.

### Pathological examination

TFE3-RCC may grossly mimic conventional clear-cell RCC in HE staining. Immunohistochemistry tests (IHC) can distinguish them from conventional clear-cell cancers. The most distinctive IHC feature of TFE3-RCC is detectable nuclear staining for chimeric (mutant) TFE3 protein, which is absent in conventional clear cells. The IHC test has a diagnostic 97.5% sensitivity and 99.6% specificity [16, 17]. Based on the unique translocation of the TFE3 gene in Xp11.2 translocation renal cell carcinoma, fluorescence *in situ* hybridization (FISH) was used to design a TFE3 gene separation probe, which was specifically bound to the DNA fragments at both ends of the target gene TFE3 to display its location and detect its TFE3 in the tissue, to diagnose TFE3-RCC further [17, 18].

Since only immunohistochemical examination is available at the Second Affiliated Hospital of Zhejiang Chinese Medical University, FISH pathological test is not routinely conducted; therefore, all indistinguishable pathological sections were sent back to the Laboratory Center of Zhejiang Provincial People’s Hospital for IHC or FISH for further diagnosis. When FISH assay findings contradicted the IHC results, a definitive diagnosis of Xp11.2 RCC was made through genetic investigation, which included FISH assays and other molecular biology.

### Baseline data collection

Regarding the nomogram, we collected baseline information from R and H cohort, which included age, sex, body mass index, personal history (smoking, drinking, heart disease, hypertension, and diabetes), and hematological indicators (MON, EOS, BAS, Fib, D-dimer, ALB, ALT, AST, GGT, BA, GLU, BUN, UA, TC, TG, HDL, LDL, and lactate dehydrogenase).

Regarding prognostic analysis, patient information was collected from the T cohort, which included age, sex, pT staging, invasion of regional lymph nodes, location of tumors, tumor boundary (Based on preoperative imaging), tumor necrosis or bleeding, immunohistochemical staining (Cathepsin-K, CD10, CK7, CA-IX, and PAX-8), surgical method, targeted medicine (mTOR inhibitors and tyrosine kinase inhibitors), progression, and prognosis.

Telephone consultation was arranged by assistants for prognostic data, and all patient information was sent to the Zhejiang Provincial People’s Hospital for secondary reviewing and grouping (Supplementary Table 1).

**Figure 1:**
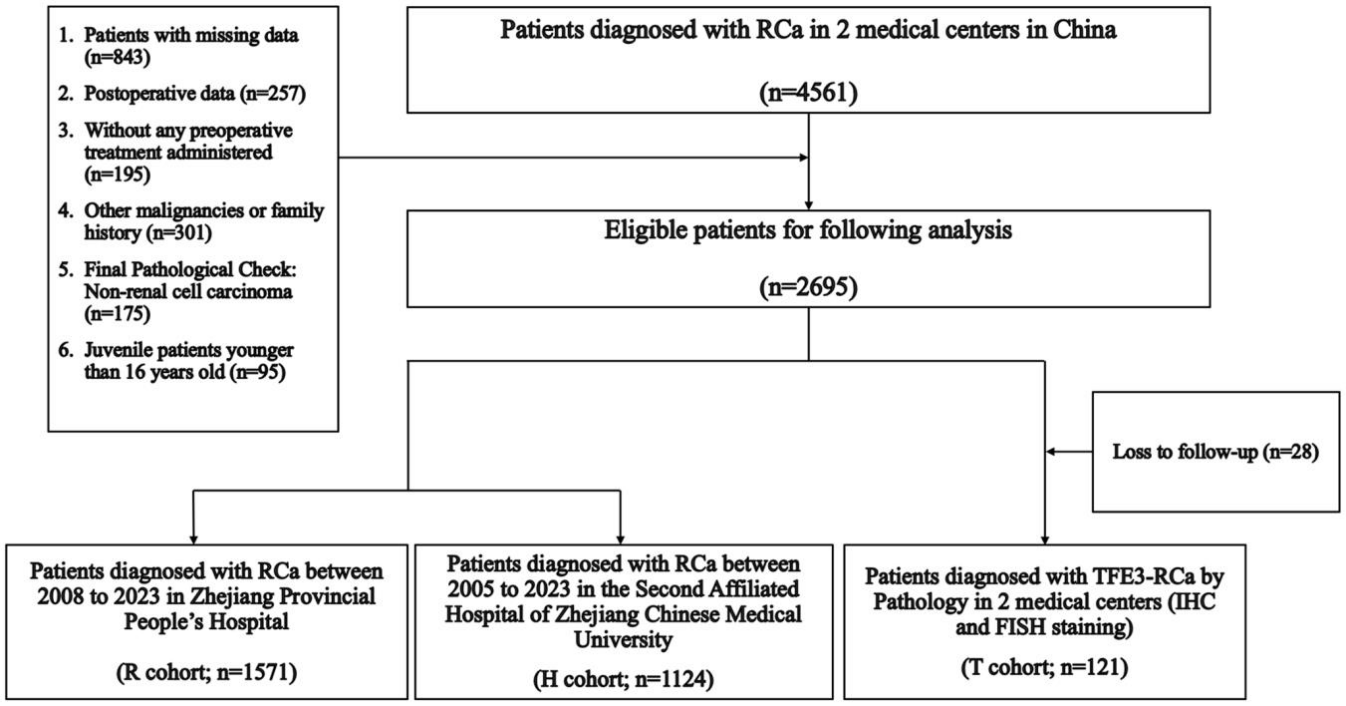
Enrollment process.

A set of inclusion and exclusion criteria was strictly formulated to screen eligible patients in two medical centers: (1) Enrolled cases with complete baseline information; (2) pathological examination: All paraffin section staining reports indicated renal cell carcinoma, and ambiguous pathological results were sent to immunohistochemical or fluorescent *in situ* hybrid (FISH) examination; (3) clinical data were collected preoperatively; (4) enrolled cases without any preoperative treatment; (5) patients with other malignancies or family history of RCa were excluded; (6) adult patients older than 16 years. The “R,” “H,” and “T” cohorts were remarked as the “Ra,” “Ha,” and “Ta” cohorts after rigorous screening above (Figure 1).

### Model construction and validation

The nomogram was established using the Ra cohort, and nomogram accuracy was verified using the Ha external validation cohort.

Univariate binary logistic regression analysis was used to evaluate different variables. Variables with *P* < 0.05 were included in multiple logistic regression analysis models, and odds ratios (OR) and 95% confidence intervals (95% CI) were also calculated. Statistically significant variables in the multivariate analysis were used to build the nomogram. The apparent performance of the nomogram was evaluated using bootstrap validation in the Ha cohort. Discrimination was measured using the C-statistic, which was equal to the area under the curve (AUC) calculated by plotting the receiver operating characteristic (ROC) curve. Finally, calibration was performed by drawing a calibration curve.

### Decision curve analysis (DCA)

A DCA curve was constructed to predict the TFE3-RCC probability. R studio was used to quantify the clinical net benefit of our model under different threshold probabilities.

### Statistical analysis

SPSS (version 26.0) and R (version 4.1.1) were used for the statistical analyses. We followed the STROBE guidelines for research and data analysis. The classification data are presented as numbers and percentages. Patient baseline characteristics are presented as means, interquartile range (IQR), numbers, and proportions. A log-rank test was performed to analyze the effects of each variable on progression and prognosis. Variables with statistical significance were included in the Multivariate COX survival regression model, and independent predictors derived from the model were described using the Kaplan-Meier survival curve. *P* < 0.05 was considered a statistically significant difference.

## RESULTS

Based on strict compliance with the inclusion criteria, 1571, 1124, and 121 patients were included in the Ra, Ha, and Ta cohorts, respectively, and all clinical parameters are summarized in Table 1. The TFE3-RCC positive rates in the R and H cohorts were 3.4% (88/2520) and 2.9% (61/2041), respectively. In this study, the sex incidence ratio was 1:1.9, and females had a considerably higher incidence than males. Age appears to be a protective factor for morbidity, and younger patients are more likely to suffer from TFE3-RCC than elder patients (HR: 5.287, *p* = 0.021).

**Table 1:** Demographic characteristics of the patients in each cohort.

**Clinical parameters**	**R (*n* = 2520)**	**H (*n* = 2041)**	**T (*n* = 149)**
	**Ra (*n* = 1571)**	**Ha (*n* = 1124)**	**Ta (*n* = 121)**
Median age, *y* (IQR)	59.9 (50.0–70.0)	60.5 (52.0–70.0)	61.9 (53.0–72.0)
Sex			
Male	720 (45.8)	587 (52.2)	42 (34.7)
Female	841 (54.2)	537 (47.8)	79 (65.3)
Median BMI, kg/m^2^ (IQR)	23.3 (21.2–25.3)	23.4 (21.3–25.5)	24.2 (21.9–26.2)
<24	954 (60.7)	654 (58.2)	52 (43.0)
≥24	617 (39.3)	470 (41.8)	69 (57.0)
Diabetes	181 (11.5)	143 (12.7)	8 (6.6)
Hypertension	646 (41.1)	496 (44.1)	44 (36.4)
Cardiac disease	85 (5.4)	71 (6.3)	8 (6.6)
Alcoholism	514 (32.7)	399 (35.5)	31 (25.6)
Smoking	530 (33.7)	344 (30.6)	71 (58.7)

Abbreviations: IQR: interquartile range; BMI: body mass index; TFE3-RCC: Xp11.2 translocation/TFE3 gene fusion renal cell carcinoma.

### Establishment of the predictable nomogram

**Figure 2:**
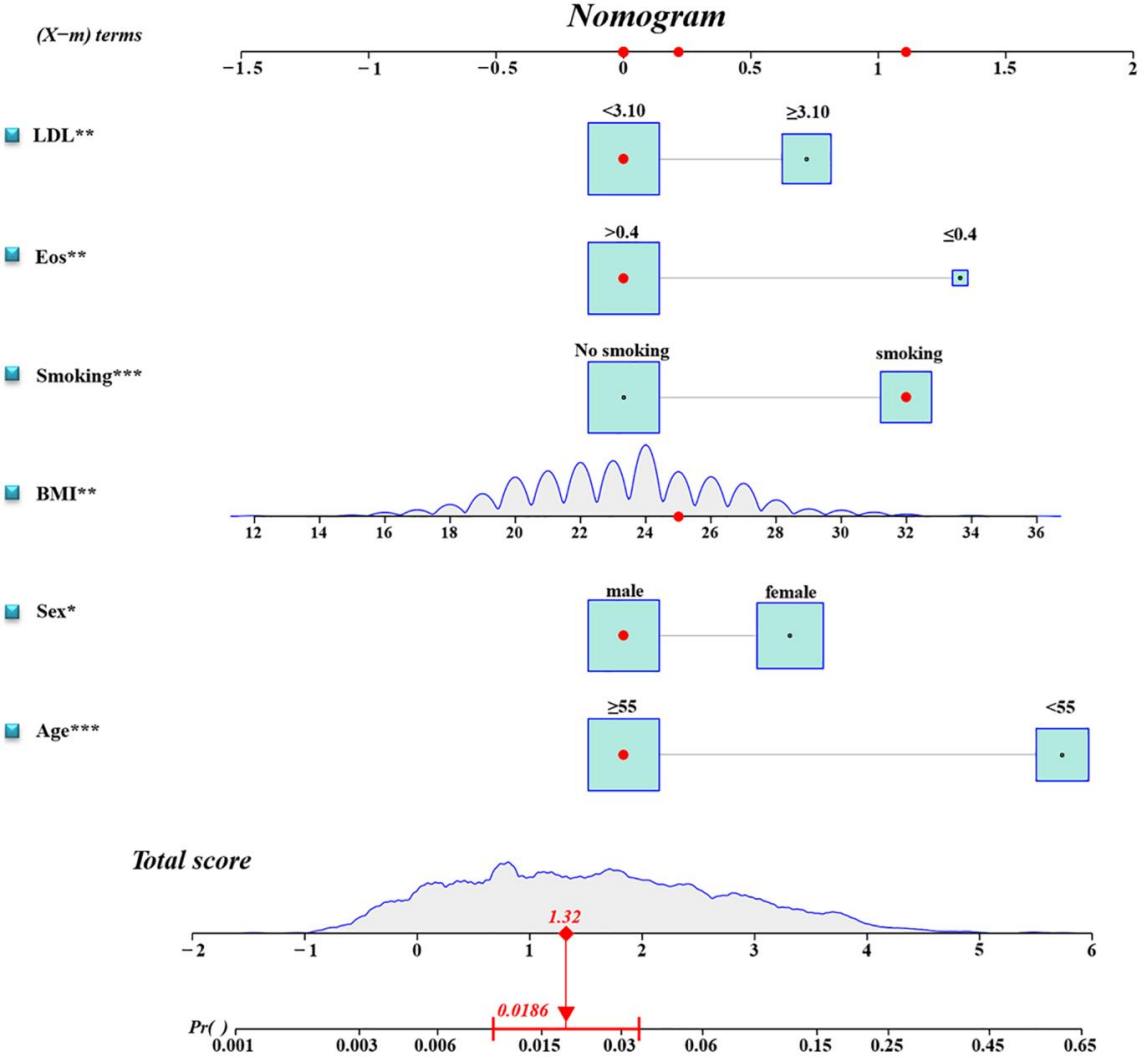
**Diagnostic nomogram.** An accurate TFE3-RCC diagnostic nomogram constructed using age, sex, BMI, smoking, eosinophil count, and LDL level.

A binary logistic regression analysis was performed to screen for predictors of RCa in the Ra cohort. Univariate analysis revealed that younger age, female, high BMI, smoking, low EOS, and high LDL were significantly linked to TFE3-RCC diagnosis (Table 2 and Supplementary Table 2). The subsequent multiple logistic regression analysis included variables related to the TFE3-RCC diagnosis in the univariate analysis (Table 2). Therefore, a predictive model containing six variables was established (Figure 2).

**Table 2:** Univariate and multivariate analysis for screening the predictors of outcomes of TFE3-RCC.

	**Univariate model**			**Multivariate model**			
	**OR**	**95% CI**	***P***	**B**	**OR**	**95% CI**	***P***
Younger age	5.305	3.095~9.095	<0.001	1.725	5.611	3.230~9.749	<0.001
Female	1.775	1.086~2.900	0.022	0.654	1.923	1.147~3.224	0.013
BMI	1.114	1.032~1.202	0.006	0.127	1.135	1.046~1.232	0.002
Smoking	2.849	1.745~4.650	<0.001	1.104	3.015	1.803~5.042	<0.001
Low EOS	3.208	1.468~7.009	0.003	1.328	3.773	1.604~8.879	0.002
High LDL	2.126	1.310~3.452	0.002	0.712	2.039	1.229~3.383	0.006

Abbreviations: BMI: body mass index; EOS: Eosinophils; LDL: Low-density lipoprotein.

**Figure 3:**
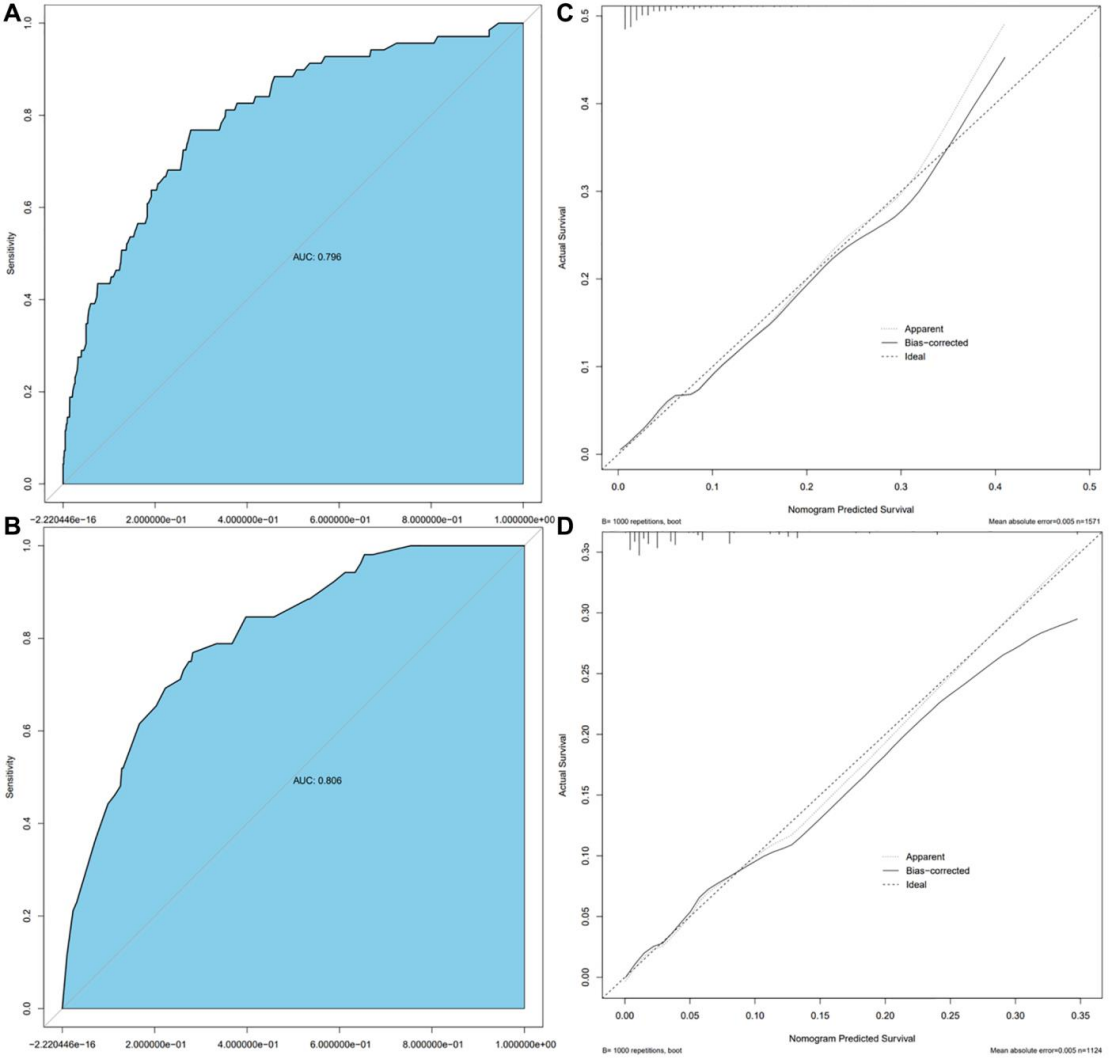
**Calibration curve and ROC curve.** Discrimination of the nomogram was evaluated by the ROC curve, AUC = 0.796 in the Ra cohort (**A**), AUC = 0.806 in the Ha cohort (**B**); calibration curves illuminate the agreement between the predicted risks of TFE3-RCC (**C**) and the observed incidence of TFE3-RCC (**D**). The dotted line represents the ideal flawless model.

Model validation demonstrated that the model had good reproducibility (AUC = 0.796). Moreover, external validation showed that the prediction nomogram had excellent discrimination ability (AUC = 0.806). In both cohorts, the calibration curves indicated a high correlation between the predicted and observed TFE3-RCC incidence (Figure 3).

### Decision curve analysis

**Figure 4:**
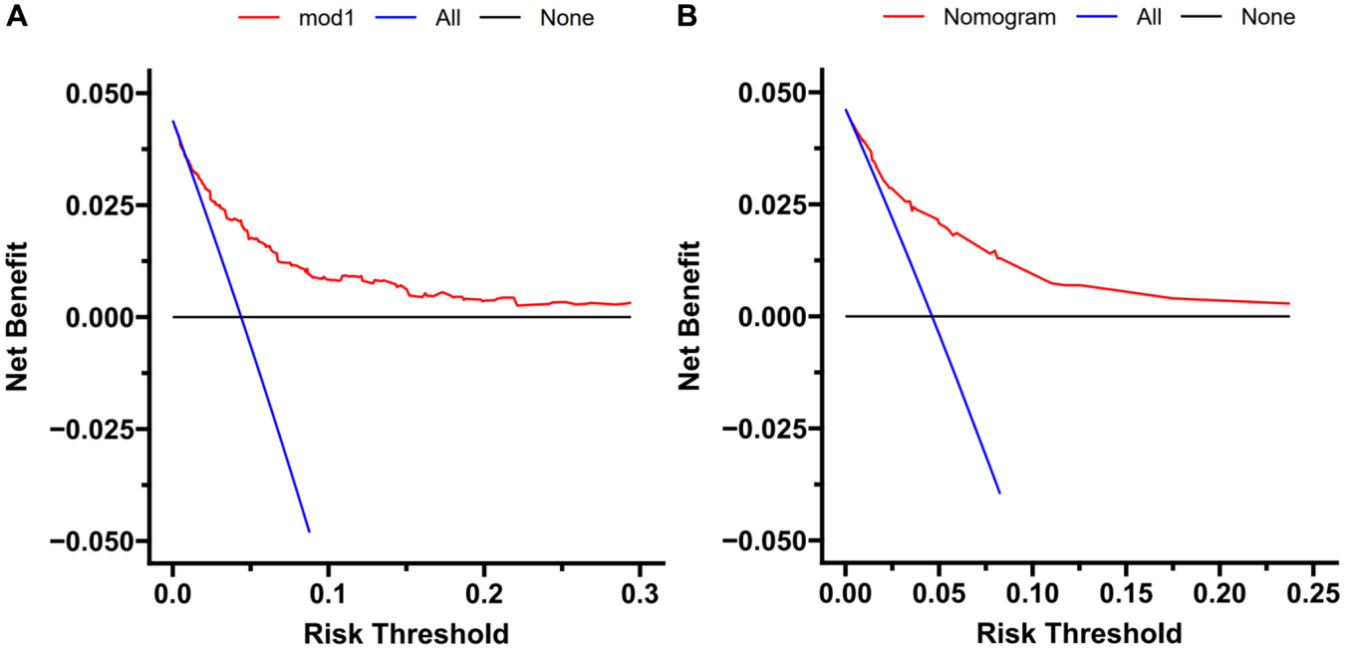
**Decision curve analysis.** DCA curve analysis of the training cohort (**A**) and external validation (**B**). Quantified net benefits were measured for different threshold probabilities. The y-axis denotes the standardized net benefit, and the x-axis denotes the threshold probabilities. The red line represents our nomogram, the blue line represents the condition of patients with TFE3-RCC, and the black line represents the condition of which none suffered from TFE3-RCC.

To evaluate the nomogram’s clinical usefulness, a DCA curve was drawn to verify that the nomogram could increase the detection rate of TFE3-RCC, which further confirmed the clinical effectiveness of the nomogram (Figure 4).

### Pathologic analysis

As for gross specimens in the Ta cohort, 84 cases showed mainly solid pattern masses, 27 displayed cystic solid masses, and 10 displayed cystic masses. Upon observing the paraffin sections, most tissue surfaces were golden yellow, but some specimens were greyish white, greyish yellow, greyish red, or multicolored.

Under the microscope, most tumor tissues revealed a nest, papillary, acinar, sheet, or mixed morphology. Most tumor cells have significant atypia, abundant and deeply stained eosinophile cytoplasm, and large nuclear kernels. Psammoma bodies can be observed in the intercellular stroma of most sections. There were 20 cases (16.5%) of tumor necrosis or hemorrhage and 37 cases (30.5%) of invasive growth.

**Figure 5:**
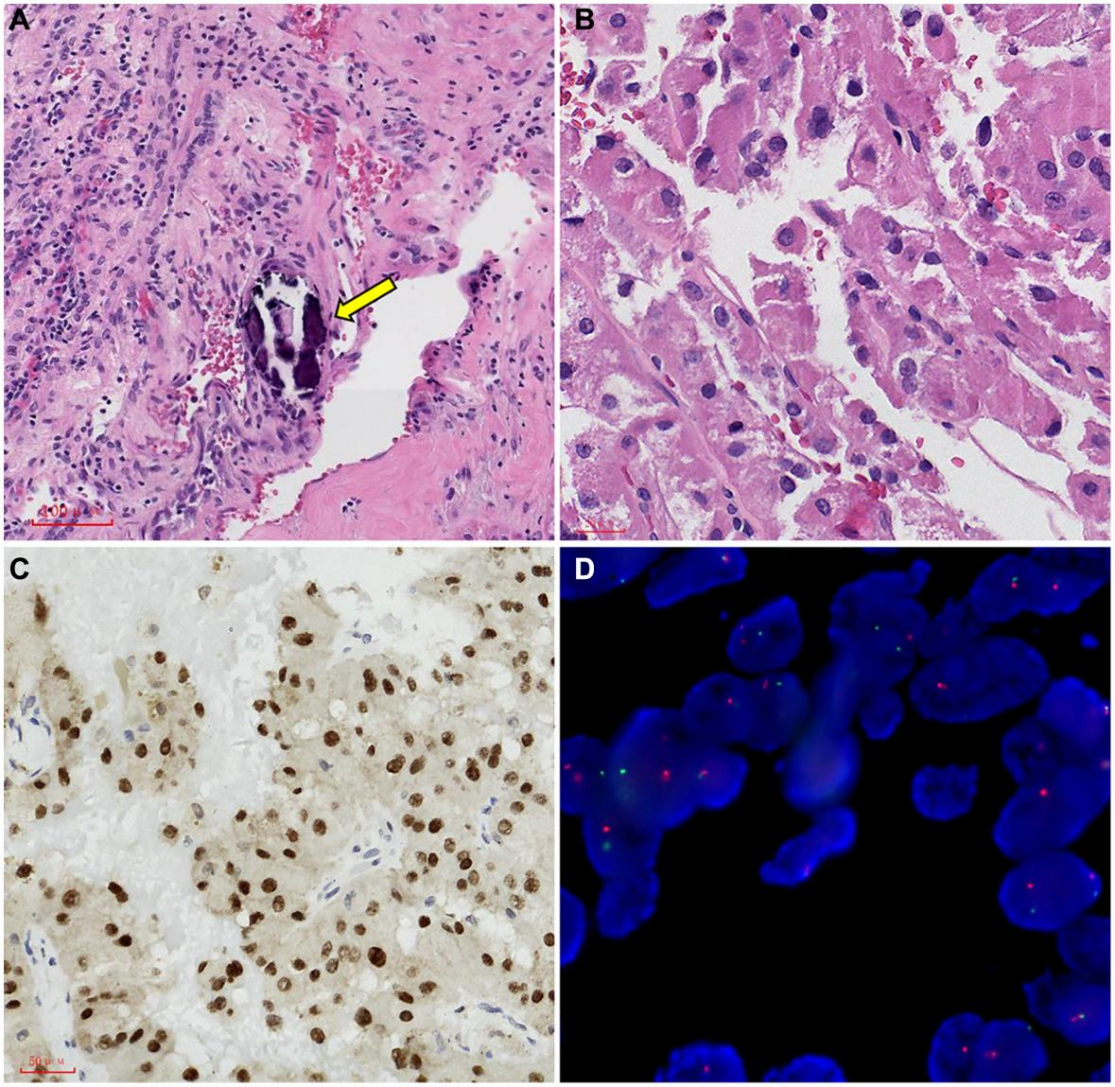
**Representative images of TFE3 immunohistochemical staining and microscopic appearance for Xp11.2 RCC.** (**A**) Blood sinusoid and Psammoma bodies were abundant in intercellular substance. The arrow points to Psammoma bodies; (**B**) abundant and deeply stained eosinophile cytoplasm, similar to renal clear cell carcinoma; (**C**) the results of immunohistochemistry showed that TFE3 was strongly positive in cancerous tissue; (**D**) FISH test results: 100 cells were counted, and the number of cells with TFE3 gene breakage was more than 20. TFE3 gene probe: Broken (positive).

All immunohistochemical tests (IHC) revealed that TFE3 was strongly diffusely positive in 121 cases, Cathepsin K was positive in 43 cases (35.5%), CD10 was positive in 30 cases (24.7%), CK7 was positive in 37 cases (30.5%), CA-X was positive in 8 cases (6.6%), and PAX8 was positive in 40 cases (33.0%) (Figure 5).

### Clinical data and prognostic analysis

After surgery, patients were reviewed every three months; the median follow-up time for 121 cases was 37 (3~96) months, and the median progression time for 38 cases with recurrence or metastasis was 12 (3~48) months. Postoperative imaging examination found that 15 cases of local recurrence, 9 cases of post-peritoneal lymph nodes were metastasized, and 14 cases were transferred in the distance (six lung metastases, four bone metastases, three liver metastases, and one cavity vein system metastasis). All patients refused to proceed with postoperative cytoreductive surgery. The progression-free survival rates at 3 and 5 years were 75.2 and 68.5%, respectively, in the Ta cohort.

“Progression” was defined as the recurrence or metastasis of the disease after surgery. Regarding disease progression, we found that stage pT1-2 patients did not benefit from different surgical modalities (HR: 0.297, 95% CI: 0.083~1.006; *P* = 0.063), while stage pT3-4 patients did benefit from processing nephrectomy rather than partial nephrectomy (HR: 0.069, 95% CI: 0.016~0.296; *P* < 0.001). The surgical methods for radical nephrectomy included five patients with lymph node dissection. Preoperative imaging revealed renal hilar lymph node invasion, and renal hilar lymph node dissection was performed intraoperatively. Five patients recovered well after surgery, and three patients received adjuvant drug therapy with TKI after surgery. Until now, none of the five patients demonstrated disease progression.

**Figure 6:**
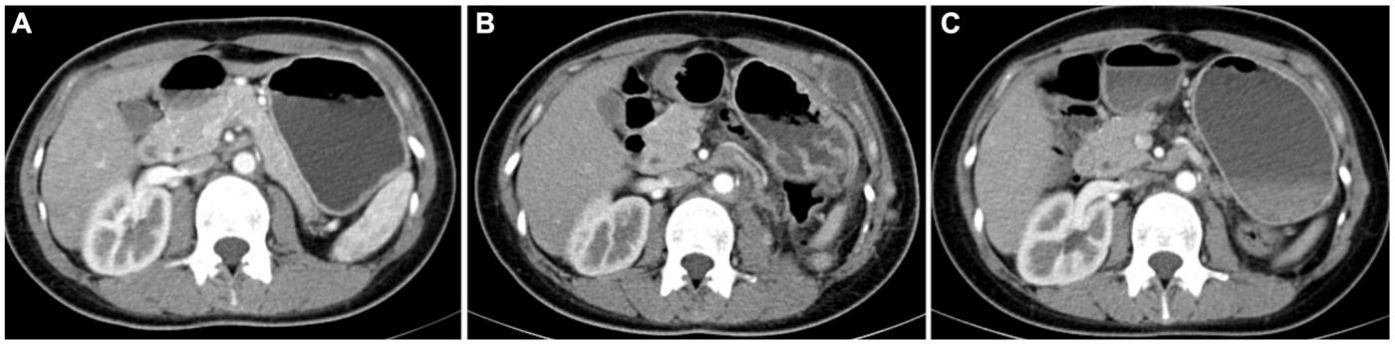
**Contrast-enhanced computed tomography results of postoperative follow-up in a patient with TFE3-RCC.** (**A**) The results of re-examination one month after the surgery; (**B**) three months after surgery, the patient’s re-examination showed multiple tumor metastases in the abdominal wall, left psoas major muscle, and pelvic cavity, so the patient started sunitinib therapy; (**C**) the re-examination results at six months after surgery showed that the metastasis was smaller than before, and the disease progression was controlled.

A total of 24 patients received postoperative medication-assisted therapy in the Ta cohort; six received mTOR inhibitor therapy, and 24 received TKI therapy. Due to TKI treatment failure, six patients chose an mTOR inhibitor. Progression was significantly controlled after the treatment procedure was replaced in five patients, while one did not experience an obvious treatment effect. Two patients were treated with a combination of TKI and mTOR inhibitor, but they abandoned the combination therapeutic procedure because of early side effects and continued the monotherapy therapeutic procedure (Figure 6).

Variables related to patient prognosis were included in the univariate analysis, which was performed using the log-rank method for inter-group comparison in the Ta cohort (Table 3).

**Table 3:** Log-rank analysis of different variables for prognosis and progression.

	***n***	**Progression**			**Prognosis**	
		**Progressive cases**	***x* ^2^**	***p*-value**	***x* ^2^**	***p*-value**
Age						
<55	26	13 (50.0)	5.287	0.021	3.027	0.082
≥55	95	25 (26.3)				
Sex						
Male	37	10 (27.0)	1.409	0.235	2.493	0.114
Female	84	28 (33.3)				
*p*T staging						
T1~2	72	14 (19.4)	5.187	0.023	5.557	0.018
T3~4	49	24 (48.9)				
Invasion of regional lymph node						
+	23	19 (82.6)	57.161	<0.001	39.003	<0.001
−	98	19 (19.4)				
Location of tumors						
Left	73	25 (34.2)	0.492	0.483	2.921	0.087
Right	48	13 (27.1)				
Tumor boundary						
Clear	84	23 (27.4)	0.812	0.368	1.356	0.244
Unclear	37	15 (40.5)				
Tumor necrosis or bleeding						
+	20	3 (15.0)	4.033	0.045	0.835	0.361
−	101	35 (34.6)				
Cathepsin-K						
+	43	14 (32.5)	0.071	0.790	0.062	0.804
−	78	24 (30.7)				
CD10						
+	30	9 (30.0)	0.005	0.944	3.640	0.056
−	91	29 (31.8)				
CK7						
+	37	16 (43.2)	2.747	0.097	1.980	0.159
−	84	22 (26.2)				
CA-IX						
+	8	1 (12.5)	0.982	0.322	0.841	0.359
−	113	37 (32.7)				
PAX-8						
+	40	11 (27.5)	0.148	0.700	<0.001	0.986
−	81	27 (33.3)				
Tyrosine Kinase Inhibitors						
+	24	16 (66.7)	39.758	<0.001	55.742	<0.001
−	97	22 (22.7)				
mTOR inhibitors						
+	6	1 (16.7)	0.273	0.601	0.646	0.422
−	115	37 (32.2)				
Surgical method						
Partial nephrectomy	52	26 (50.0)	22.079	<0.001	14.825	<0.001
Radical nephrectomy	69	12 (17.4)				

Age, pT staging, regional lymph node invasion, tumor necrosis or bleeding, tyrosine kinase inhibitors, and surgical methods significantly affected the progression outcomes. pT staging, regional lymph node invasion, tyrosine kinase inhibitors, and surgical method significantly affected the prognosis.

**Figure 7:**
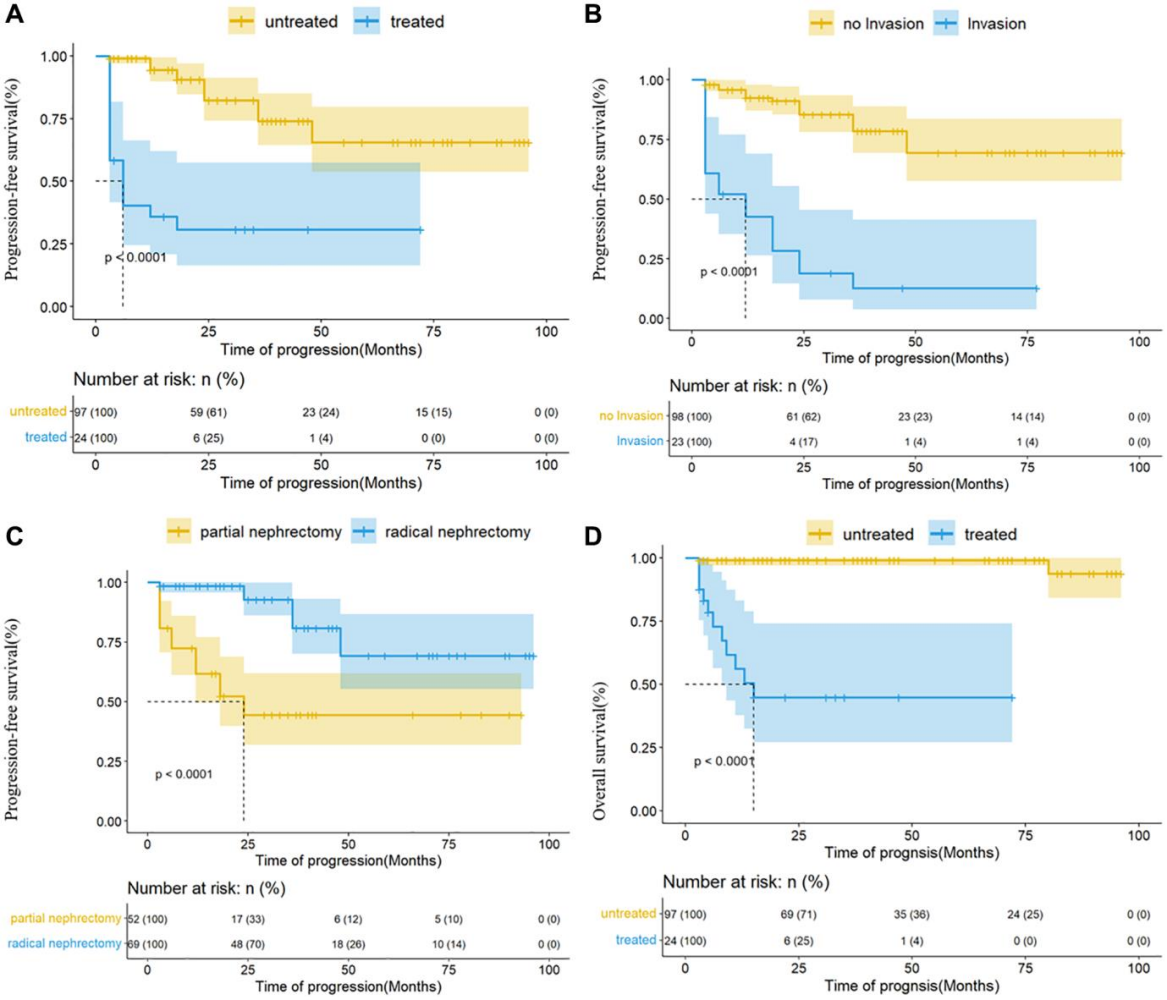
**Kaplan-Meier analysis for progression-free survival and overall survival in the Ta cohort.** (**A**) The effect of TKI on progression; (**B**) the effect of regional lymph node invasion on progression; (**C**) the effect of surgical methods on progression; (**D**) the effect of TKI on prognosis.

Subsequently, the variables mentioned above were subsumed within the multi-factor model, and the results depicted that invasion of regional lymph nodes, tyrosine kinase inhibitors, and surgical methods were independent factors related to progression, while tyrosine kinase inhibitors were also associated with prognosis (Table 4 and Figure 7).

**Table 4:** Multivariate COX survival regression analysis of prognosis and progression.

	**Progression**			**Prognosis**		
	**HR**	**95% CI**	***p*-value**	**HR**	**95% CI**	***p*-value**
Age	1.444	0.701~2.976	0.319	–	–	–
*p*T staging	1.793	0.840~3.827	0.131	3.191	0.814~12.510	0.096
Invasion of regional lymph node	4.326	1.942~9.641	<0.001	3.013	0.737~12.324	0.125
Tumor necrosis or bleeding	0.398	0.111~1.430	0.158	–	–	–
Surgical method	0.374	0.163~0.856	0.020	0.165	0.019~1.397	0.098
Tyrosine Kinase Inhibitor	3.217	1.489~6.949	0.003	25.121	2.955~13.554	0.003

## DISCUSSION

Renal cell carcinoma is the sixth most prevalent cancer in males (5%) and the ninth in women (3%) by 2022. In contrast to the rapidly declining incidence of prostate cancer, the incidence of renal cell carcinoma seems to be increasing yearly [19]. The 2016 WHO Histological Classification of Renal Tumors classifies renal cell carcinoma into 16 subtypes; one subtype is Mit family translocation RCC [8], which mainly consists of Xp11.2 translocation /TFE3 gene fusions RCC, TFEB/t (6;11) translocation RCC, and MITF translocation RCC [20, 21], which share similar clinical features, histological, immunohistochemical, and molecular genetic characteristics, but are significantly different from other RCC. Among MITF family members, TFE3 translocations are the most prevalent [8, 22], and many cases of TFE3-RCC have been reported in recent years. According to previous studies, TFE3-RCC has strong invasion, rapid progression, and poor prognosis compared to common types of renal cell carcinoma [13–15, 22, 23]. Clinicians and pathologists lack awareness of its pathology and clinical presentation due to the paucity of case reports of TFE3-RCC, and the possibility of missed or delayed diagnosis cannot be ruled out. Therefore, it gives great clinical significance to diagnose TFE3-RCC and formulate an effective treatment plan early.

In this study, we built a prediction model for TFE3-RCC using age, sex, BMI, smoking, eosinophils, and low-density lipoprotein as predictors. The model was externally validated and demonstrated high predictability.

We found that BMI and LDL were strongly associated with early predictions of TFE3-RCC. Previous studies have revealed that obesity may affect RCC risk through multiple mechanisms, and adipose tissue appears to regulate TFE3-RCC risk [24]. Because adipose tissue is an important endocrine gland that synthesizes and secretes numerous hormones and cytokines (adipokines), studies have revealed that obesity reduces serum adiponectin levels. Adiponectin shows anti-tumor activity because of its anti-inflammatory and anti-proliferative effects and its antagonistic effect on insulin [25–29]. Therefore, decreased adiponectin levels are more likely to lead to tumor growth. Moreover, *in vitro* studies have confirmed that adiponectin can inhibit tumor growth by activating AMP-activated protein kinase (AMPK) and inhibiting the mammalian rapamycin target (mTOR) pathway, validating the role of obesity in TFE3-RCC [30–32]. Therefore, it is correct to choose BMI and LDL levels as predictors in the prediction model.

Prognostic data were collected and analyzed for the Ta cohort. There was no statistically significant difference between radical and partial nephrectomy in TFE3-RCC patients with stage pT1~2 disease. Therefore, surgical plans could be made for these patients based on factors such as tumor size and technical support. However, we strongly recommend radical nephrectomy if pT3-4 patients are indicated for surgery, which may prevent carcinoma progression. Although some studies have claimed that TFE3-RCC is an inert renal cell carcinoma [11, 12], 38 patients (31.4%) in this Ta cohort had disease progression, and most patients were prone to local recurrence *in situ*, lung metastasis, and bone metastasis; accordingly, postoperative review is particularly critical. Preoperative imaging or intraoperative local lymph node invasion suggests tumor progression risk. Given the positive impact of local lymph node invasion on tumor progression, we strongly recommend that surgeons perform regional lymph node dissection for such TFE3-RCC patients.

Due to the high concentration of MDR-1 (drug-resistant gene) in renal carcinoma cells, its product P-glycoprotein (p170) can pump chemotherapeutic drugs out of tumor cells; thus, traditional chemotherapy drugs are mostly ineffective for renal carcinoma [33]. TKI are first-line treatment drugs that have an obvious therapeutic effect on TFE3-RCC [34, 35]. Although TKI therapy may fail, it can postpone disease progression and is the only factor that determines patient prognosis. An mTOR inhibitor is the second-line treatment [36]. Although log-rank analysis displayed no statistically significant effect of mTOR inhibitors on patient prognosis and disease progression, we believe that mTOR inhibitors can effectively prevent TFE3-RCC progression, which may also be due to the lack of data. Therefore, patients were highly recommended to use targeted drugs after surgery (TKI and mTOR inhibitors) to avoid further disease progression.

We used early prediction models, therapy options, and prognosis risk factors to diagnose and treat TFE3-RCC. This study also has several limitations: (1) The patients included were all from Zhejiang Province, China. A more accurate prediction model and convincing prognostic analysis may require multi-center data support in other provinces or countries. (2) For TFE3-RCC patients, the comparison between treatment options was limited to surgical modalities and postoperative targeted drug therapies. (3) Whether drug selection for systematic therapy is effective is unclear. Individual gene detection programs are highly recommended; however, these are still limited by the lack of popularity of genetic testing in China. (4) Due to the absence of a database, many indicators, such as tumor indicators, do not participate in the composition of the prediction model. (5) This study’s database supports the research conclusions, but larger sample databases and prospective investigations are needed to test the predictive model’s accuracy and predictability.

## CONCLUSION

In this study, six variables (age, sex, BMI, smoking, eosinophils, and LDL) were used to create an accurate prediction model for TFE3-RCC, which provided a calculator for early diagnosis by clinicians. Moreover, studies have found that surgical strategy, postoperative targeted drug therapy, and local lymph node invasion are important factors associated with progression in TFE3-RCC patients, and TKI plays an important role in prolonging the survival of patients; therefore, clinicians can consider applying TKI consolidation therapy to postoperative patients.

## Supplementary Materials

Supplementary Tables
