# Supplementary material for: Therapeutic strategies and predictive models for Xp11.2 translocation/TFE3 gene fusion renal cell carcinoma in adults based on data of two Chinese medical centers
Source: Aging (Albany NY). 2024 Jan 22;16(2):1696–711. doi: 10.18632/aging.205452 (PMC10866448; doi:10.18632/aging.205452)
Supplement: Supplementary Tables [file aging-16-205452-s001.pdf]

SUPPLEMENTARY TABLES

Supplementary Table 1. Grouping criteria.

	Low-group	Standard group	High-group
BMI (kg/m^2)	–	<24.00	≥24.00
Mon (10^9/L)	–	<0.6	≥0.6
Bas (10^9/L)	–	<0.06	≥0.06
Eos(10^9/L)	≤0.4	>0.4	–
Fib (g/L)	–	<4	≥4
D-Dimer (ug/L)	–	<550	≥550
ALB(g/L)	≤40.00	>40	
ALT(U/L)	–	<40	≥40
AST(U/L)	–	<35	≥35
AUT (U/L)		<45	≥45
BA (umol/L)	–	<15.00	≥15.00
GLU (umol/L)	–	<6.16	≥6.16
BUN (mmol/L)	–	<8.80	≥8.80
UA (umol/L)	–	<357.00	≥357.00
TC (mmol/L)	–	<5.96	≥5.96
TG (mmol/L)	–	<1.70	≥1.70
HDL (mmol/L)	≤1.10	>1.10	–
LDL (mmol/L)	–	<3.10	≥3.10
LDH (U/L)	–	<250.00	≥250.00

Grouping criteria for each variable.

Supplementary Table 2. Binary logistic regression analysis.

0	B	SE	Wald	df	*p*	EXP	95% CI	
							Floor	Upper
Age	1.669	0.275	36.817	1	0.000	5.305	3.095	9.095
Sex	−0.574	0.251	5.242	1	0.022	0.563	0.345	0.921
Diabetes	−0.530	0.471	1.265	1	0.261	0.589	0.234	1.482
Hypertension	−0.418	0.264	2.510	1	0.113	0.659	0.393	1.104
Cardiac disease	−0.673	0.726	0.858	1	0.354	0.510	0.123	2.119
Alcoholism	−0.414	0.285	2.113	1	0.146	0.661	0.378	1.155
Smoking	1.047	0.250	17.531	1	0.000	2.849	1.745	4.650
BMI	0.108	0.039	7.661	1	0.006	1.114	1.032	1.202
Bas	−0.145	0.602	0.058	1	0.809	0.865	0.266	2.815
Eos	1.166	0.399	8.542	1	0.003	3.208	1.468	7.009
Fib	0.492	0.353	1.942	1	0.163	1.636	0.819	3.269
D-Dimer	0.471	0.252	3.506	1	0.061	1.602	0.978	2.622
ALB	0.156	0.250	0.391	1	0.532	1.169	0.717	1.906
ALT	−0.491	0.471	1.084	1	0.298	0.612	0.243	1.542
AST	−0.413	0.405	1.040	1	0.308	0.661	0.299	1.464
GGT	−0.050	0.306	0.026	1	0.871	0.952	0.522	1.733
BA	−18.174	4641.084	0.000	1	0.997	0.000	0.000	0.000
GLU	0.066	0.299	0.049	1	0.825	1.068	0.595	1.918

BUN	−0.743	0.404	3.376	1	0.066	0.476	0.216	1.051
UA	−0.041	0.247	0.028	1	0.867	0.960	0.592	1.557
TC	−0.012	0.408	0.001	1	0.977	0.988	0.444	2.197
TG	0.260	0.262	0.985	1	0.321	1.297	0.776	2.166
HDL	0.250	0.246	1.028	1	0.311	1.284	0.792	2.081
LDL	0.754	0.247	9.319	1	0.002	2.126	1.310	3.452
LDH	0.300	0.440	0.465	1	0.495	1.350	0.570	3.195

The result of each variable in the logistic regression analysis.
